# The humanized BLT mouse to study HIV transmission

**DOI:** 10.1186/1742-4690-9-S2-P197

**Published:** 2012-09-13

**Authors:** M Deruaz, TT Murooka, T Dudek, VD Vrbanac, T Tivet, KC Bankert, TM Allen, AM Tager, AD Luster

**Affiliations:** 1 Massachusetts General Hospital, Harvard Medical School, Charlestown, MA, USA; 2 Ragon Institute of MGH, MIT and Harvard, Charlestown, MA, USA

## Background

Worldwide, the majority of HIV-1 infections are acquired by vaginal transmission. Studies in SIV-1 infected non-human primates have shown that SIV-1 infection takes hold initially in a small population of CCR5+ cells in the in female lower genital tract (FLGT) where the infection expands first locally before disseminating to the draining lymph node (LN) to establish a systemic infection. Our goal is to use humanized BLT mice to address whether the infection paradigm established for SIV-1 in non-human primates holds true during HIV-1 infection in vivo.

## Methods

We studied the kinetics of infection during the first two weeks after intravaginal HIV-1 exposure by measuring the presence of virus in the FLGT, LNs and blood after 2, 6, 10 and 12 days post infection (p.i.) by qPCR and flow cytometry.

## Results

Our results show that similar to the non-human primate model, the presence of virus is first detected by qRT-PCR in the FLGT as soon as day 2 p.i., followed by the LN at day 6 p.i. and the blood at day 12 p.i.. Similar but delayed kinetics were observed using p24 staining by flow cytometry, with positive staining of T cells located in the FLGT at day 6 p.i., in the draining LN between day 6 and day 10, and in the non draining LN at day 12 p.i..

## Conclusion

Our data suggests that HIV-1 transmission and initial replication in BLT mice following intravaginal exposure occurs first locally in the LGT and then disseminates to the draining LN. The virus then spreads to the non-draining LNs and subsequently into the blood, suggesting that BLT mice have an “eclipse phase” following HIV infection similar to what have been described for SIV infection of macaques and HIV infection of humans.

